# Supplementary material for: Comprehensive Profiling of Mammalian Tribbles Interactomes Implicates TRIB3 in Gene Repression
Source: Cancers (Basel). 2021 Dec 16;13(24):6318. doi: 10.3390/cancers13246318 (PMC8699236; doi:10.3390/cancers13246318)

**Supplementary Figure S1**

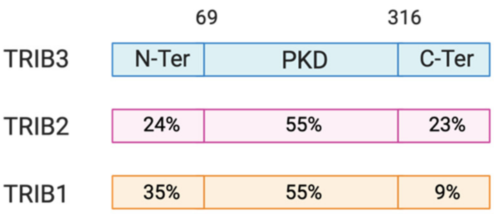


Supplementary Figure S1: Comparison of human TRIB1/2 and 3 protein sequence using ClustalOmega software.

**Supplementary Figure S2**

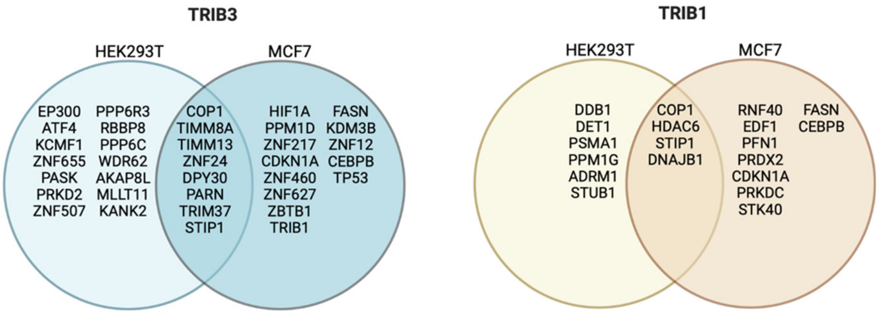


Supplementary Figure S2: Comparison of TRIB1 and TRIB3 interactors in HEK293T andMCF7 cells.

**File S1: TRAIN Consortium Authorship:**

| **Surname** | **First Name** | **Institution** |
|---|---|---|
| Bhutia | Kunzangla | Instituto de Investigacion Santaria, Hospital Clinico SanCarlos |
| Brouard | Sophie | University of Nantes |
| Barril | Xavier | University of Barcelona |
| Carracedo | Arkaitz | Asociación Centro de Investigación Cooperativa en Biociencias |
| Castillo Lluva | Sonia | Instituto de Investigacion Santaria, Hospital Clinico SanCarlos |
| Danger | Richard A.E.D | University of Nantes |

| | | |
|---|---|---|
| Day | Jack | Instituto de Investigacion Santaria, Hospital Clinico SanCarlos |
| Deshmukh | Sumeet R | The University of Sheffield |
| Feseha | Yodit | University of Nantes |
| Francis | Sheila | The University of Sheffield |
| Grzesik | Dominika J | William Harvey Research Institute, Queen Mary University, London |
| Hernandez Quiles | Miguel | Universitair Medisch Centrum Utrecht |
| Kalkhoven | Eric | Universitair Medisch Centrum Utrecht |
| Kiss-Toth | Endre | The University of Sheffield |
| Linford | Adam J | Institute for Diabetes and Cancer (IDC), Helmholtz Zentrum München |
| Martinez Campesino | Laura | The University of Sheffield |
| Metherall | Louise A | William Harvey Research Institute, Queen Mary University, London |
| Morris | Imogen | Universitair Medisch Centrum Utrecht |
| Niespolo | Chiara | The University of Sheffield |
| Pellegata | Natalia S | Institute for Diabetes and Cancer (IDC), Helmholtz Zentrum München |
| Ruiz Cantos | Miriam | William Harvey Research Institute, Queen Mary University, London |
| Salamanca Viloria | Juan | University of Barcelona |
| Satam | Swapna S | Institute for Diabetes and Cancer (IDC), Helmholtz Zentrum München |
| Scheideler | Marcel J.C. | Institute for Diabetes and Cancer (IDC), Helmholtz Zentrum München |

| | | |
|---|---|---|
| Shahrouzi | Parastoo | Asociación Centro de Investigación Cooperativa en Biociencias |
| Shologu | Ziyanda | Universidade da Beira Interior |
| Shoulders | Carol C | William Harvey Research Institute, Queen Mary University, London |
| Sudbery | Ian | The University of Sheffield |
| Socorro | Silvia | Universidade da Beira Interior |
| Velasco | Guillermo | Instituto de Investigacion Santaria, Hospital Clinico SanCarlos |
| Villacanas Perez | Oscar | MindtheByte Ltd |
| Wilson | Heather | The University of Sheffield |

File S2: Original Western blot figures

Original western blot figures of Figure 1: Co-IP Trib3 & ZBTB1

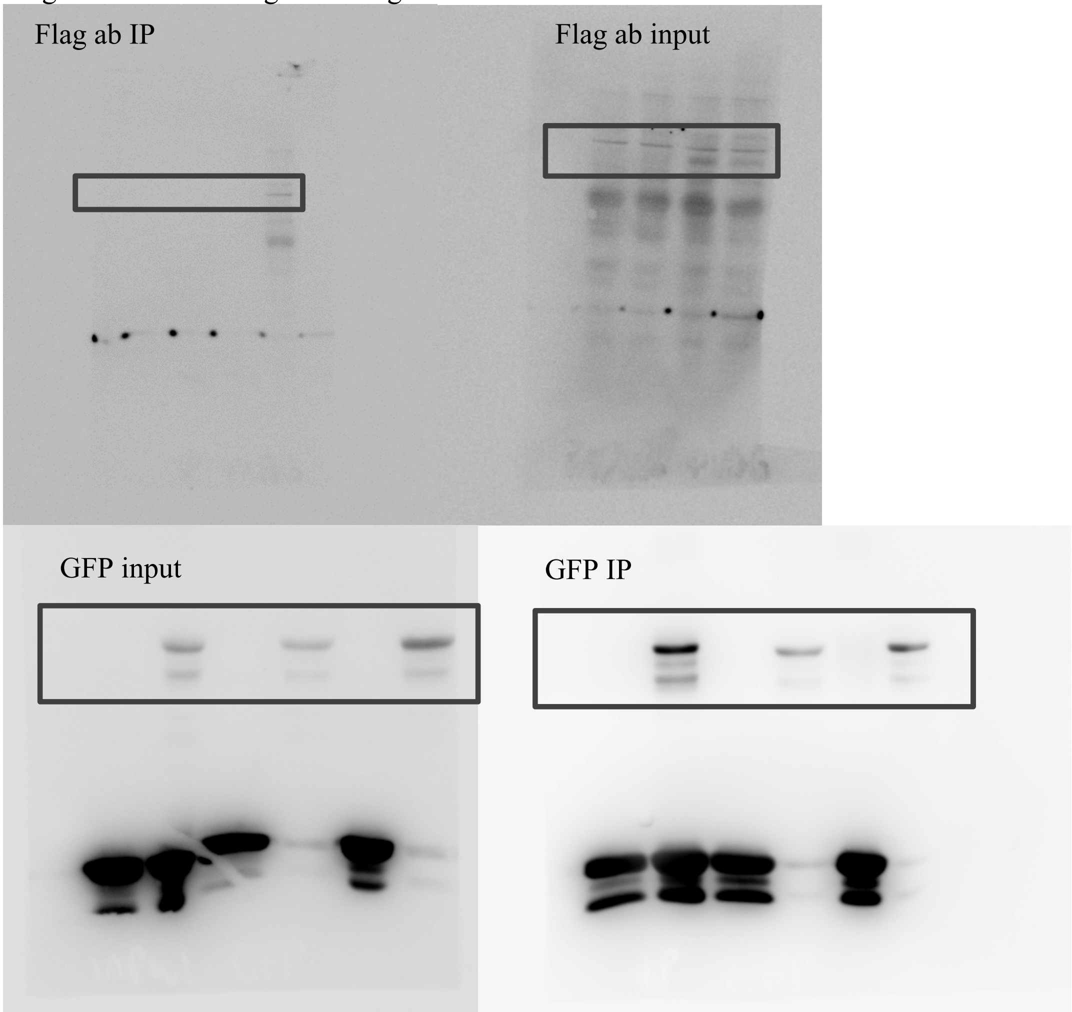

Original western blot figures of Figure 2: Gal4DBD & Tubulin ab

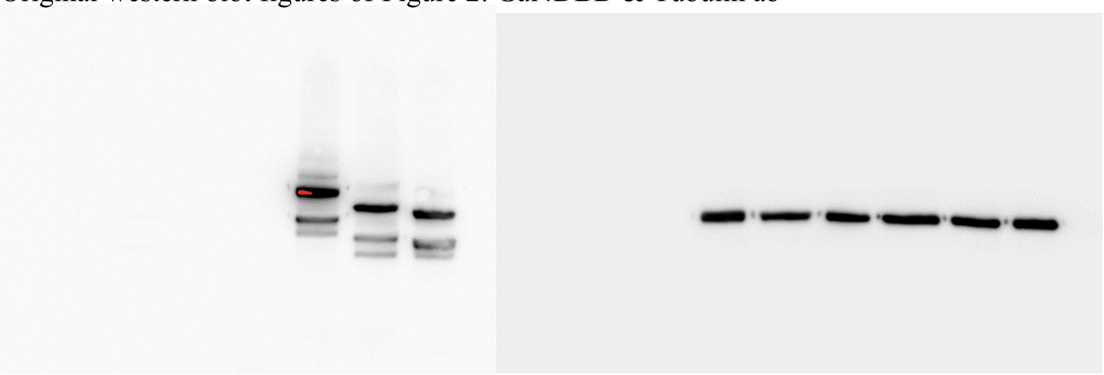

Original western blot figures of Figure 3: tGFP ab & tubulin MCF7

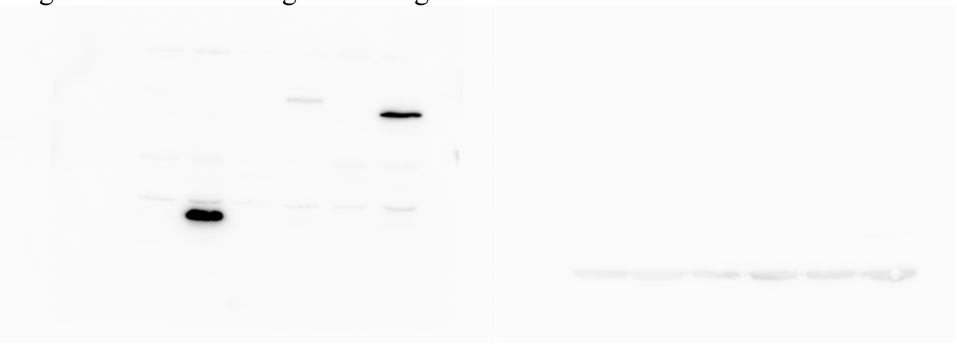

Supplement: Supplementary file 1 [file cancers-13-06318-s001.zip › cancers-1469610-supplementary.pdf]
